# Genotypic and phenotypic features of 23 Egyptian patients with tuberous sclerosis complex

**DOI:** 10.1186/s12887-026-07095-9

**Published:** 2026-06-10

**Authors:** Amr A. Othman, Abdelrahim A. Sadek, Mohammed A. Aladawy, Shereen Philip Aziz, Ahmed Sedky, Tarek M.M. Mansour, Mohammed M.S. Younis, Rania G. Abdelatif, Abd El-Monem M. Hassan, Marwa Ali Aboelmagd, Marwa Mohamed, Kawashty R. Mohamed, Salma Mohamed Elkousy, Nada M. Montaser Elkady, Rin Khang, Seung Woo Ryu, Elsayed Abdelkreem

**Affiliations:** 1 Neuropsychiatry Unit, Department of Pediatrics, Faculty of Medicine, Sohag University, Sohag, Egypt; 2 Neuropsychiatry Unit, Department of Pediatrics, Faculty of Medicine, Al- Azhar University, Assiut, Egypt; 3 Department of Clinical and Chemical Pathology, Faculty of Medicine, Sohag University, Sohag, Egypt; 4 Department of Radio-diagnosis, Faculty of Medicine, Al-Azhar University, Assiut, Egypt; 5 Department of Pediatrics, Faculty of Medicine, Al-Azhar University, Assiut, Egypt; 6 Department of Pediatrics, Faculty of Medicine, Sohag University, Sohag, 82524 Egypt; 7 Department of Dermatology, Venereology and Andrology, Faculty of Medicine, Sohag University, Sohag, Egypt; 8 Department of Neurology, Faculty of Medicine, Al-Azhar University, Assiut, Egypt; 9 Department of Radiology, Sohag General Hospital, Sohag, Egypt; 10 Medical Genetics Division, 3billion Inc., Seoul, South Korea; 11 Department of Clinical Sciences, Al-Rayan National College of Medicine, Al-Rayan National Colleges, Al Madinah Al Munawwarah, Saudi Arabia

**Keywords:** *TSC1*, *TSC2*, Neurocutaneous syndrome, Whole-exome sequencing, Egypt

## Abstract

**Background:**

Tuberous sclerosis complex (TSC) is a multisystem disease caused by pathogenic variants in *TSC1* or *TSC2* genes. Although features of TSC have been described in various populations, genetic data from Egypt remain scarce. This study aimed to characterize the phenotypic and molecular features of TSC among a cohort of Egyptian patients.

**Methods:**

This observational study included patients clinically diagnosed with TSC at two Egyptian centers between 2022 and 2025. All participants underwent a comprehensive evaluation, including demographic profiling, clinical assessment, imaging studies, and whole-exome sequencing. Identified *TSC1* and *TSC2* variants were cross-referenced with public databases, analyzed using bioinformatics tools, and classified according to the American College of Medical Genetics and Genomics guidelines.

**Results:**

The cohort contained 23 cases from 20 unrelated families (16 males and 7 females; median age: 8.1 years). Parental consanguinity and positive family history were present in 13 and 12 cases, respectively. The median age at initial presentation was 8 months (interquartile range: 3–24 months). Patients exhibited various manifestations, including hypomelanotic macules (100%), cortical tubers (96%), seizures (87%), TSC-associated neuropsychiatric disorders (78%), facial angiofibromas (65%), shagreen patches (48%), renal angiomyolipomas (44%), and cardiac rhabdomyomas (39%). Eighteen distinct variants were identified (16 in *TSC2*, 2 in *TSC1*), including nine novel variants. These comprised nine deletions/insertions, five splice-site, three nonsense, and one missense variant. Most variants (89%) were “private”, each observed in a single family.

**Conclusion:**

This study provides the first comprehensive genetic analysis of TSC in Egypt. The findings expand the demographic, phenotypic, and genetic spectrum of TSC in an underrepresented population.

**Supplementary Information:**

The online version contains supplementary material available at 10.1186/s12887-026-07095-9.

## Background

Tuberous sclerosis complex (TSC) represents an autosomal dominant disease characterized by the formation of disorganized dysplastic tissues (hamartomas) across multiple organ systems, particularly the brain, skin, kidneys, heart, and eyes [1, 2]. Approximately two-thirds of cases occur sporadically, while the remainder follows a familial inheritance pattern [3]. TSC is the second most common neurocutaneous disease, with an estimated incidence of 1 in 6,000 to 10,000 live births [1, 4]. This disease is caused by heterozygous pathogenic variants in the *TSC1* (MIM *605284, **#**191100) or *TSC2* (MIM *191092, #613254) genes, encoding hamartin and tuberin, respectively. These proteins form a heterodimeric complex with TBC1D7 that functions as a negative regulator of the mechanistic target of rapamycin (mTOR) [3, 5]. Loss-of-function variants in these tumor suppressor genes result in constitutive activation of mTOR signaling, with subsequent uncontrolled cell growth (hamartoma formation), disrupted cellular metabolism, and impaired synaptogenesis [4, 6].

Clinical manifestations of TSC are highly variable and evolve over time [5, 7, 8]. The central nervous system (CNS) is typically affected, leading to seizures, intellectual disability, and diverse TSC-associated neuropsychiatric disorders (TANDs) [6]. Structural brain abnormalities include cortical tubers, subependymal nodules (SENs), and subependymal giant cell astrocytoma (SEGA) [6]. Among cutaneous features, hypomelanotic macules usually appear in infancy, whereas facial angiofibromas tend to arise near puberty [7]. Renal angiomyolipomas (AMLs) and cysts often become apparent after puberty [9], while cardiac rhabdomyomas typically develop *in utero* and regress spontaneously after birth [5, 10]. Pulmonary lymphangioleiomyomatosis (LAM) primarily affects females over 30 years of age [7]. Brain tumors and renal complications remain the leading causes of morbidity and mortality [6].

Diagnosis of TSC relies on clinical criteria and/or the identification of a pathogenic variant in *TSC1* or *TSC2* [1, 2]. *TSC1* is composed of 23 exons (the first two being non-coding) in a 3.4-kb coding region, spanning about 50 kb on chromosome 9q34.13; exons 5 and 12 are alternatively spliced [4]. *TSC2* contains 42 exons (exon 1 is non-coding) within a 5.4-kb coding region that spans approximately 40 kb on chromosome 16p13.3 [3]. Genetic testing reveals pathogenic variants in about 85% of patients with a clinical diagnosis of TSC [6, 11, 12]. Thousands of variants have been reported, but none accounts for more than 5% of cases, which complicates genotype-phenotype correlations [4–6]. Nonetheless, *TSC2* variants occur more frequently among sporadic cases and are associated with more severe phenotypes [11, 13, 14].

Several genetic studies have been conducted in populations from Europe, North America, and Asia, significantly advancing our understanding of TSC [11, 12, 15–29]. However, there remains a notable paucity of genetic data from the Middle East and North Africa (MENA) region, including Egypt. Although some studies and case reports have described clinical and neuroimaging characteristics in Egyptian patients with TSC, only one small study utilized Sanger sequencing in five infants with cardiac rhabdomyomas [30–35]. To date, no comprehensive molecular analyses on TSC have been published from Egypt.

To address this knowledge gap, the present study aimed to characterize the phenotypic and molecular features of TSC in a cohort of 23 Egyptian patients. Our findings expand the existing demographic, clinical, and genetic spectrum of TSC in this underrepresented population.

## Materials and methods

### Study design and setting

This observational study was conducted at the Pediatric Neurology Units of Sohag and Al-Azhar (Assiut branch) University Hospitals in southern Egypt from June 2022 to May 2025. Eligibility criteria were patients with a clinical diagnosis of TSC based on the 2021 updated international diagnostic criteria, requiring either two or more major features or one major and two or more minor features [1]. Patients with coexistent acquired neurological diseases, such as perinatal asphyxia, traumatic brain injury, or CNS infections, were excluded. The study protocol was approved by the Medical Research Ethics Committee of the Faculty of Medicine, Sohag University (Approval No. Soh-Med-22-06-28). Written informed consent was obtained from the parents or legal guardians of all participants. All study procedures adhered to the ethical principles outlined in the 1964 Declaration of Helsinki and its subsequent amendments. The manuscript adheres to the Strengthening the Reporting of Observational Studies in Epidemiology (STROBE) guidelines (Additional file 1).

### Clinical, neurological, and imaging evaluations

Patients underwent a thorough clinical assessment, including detailed history taking, physical examination, and neurodevelopmental evaluation. Collected data included demographic characteristics, family history of TSC, age at symptom onset, clinical manifestations, imaging findings, and treatment history. Anthropometric measurements were assessed using the Egyptian Child Growth Standards (Diabetes Endocrine Metabolism Pediatric Unit, Cairo University Children Hospital, 2008 release; http://dempuegypt.blogspot.com). Neurodevelopmental status was evaluated using the Denver II developmental screening test (http://denverii.com). Seizures were defined according to the 2025 criteria of the International League Against Epilepsy [36]. Patients were screened for neuropsychiatric disorders using the TAND checklist [37]. Intellectual disability and behavioral disorders were diagnosed following the criteria outlined in the Diagnostic and Statistical Manual of Mental Disorders, 5th Edition (DSM-5) [38]. Dermatological evaluation was performed by expert dermatologists to characterize cutaneous features.

All patients underwent brain magnetic resonance imaging (MRI) as the gold-standard modality for TSC diagnosis and surveillance. Brain computed tomography (CT) was also performed in all patients as an initial diagnostic tool for seizures and developmental delay, particularly in emergency settings or when MRI was inaccessible or not feasible. Additional assessments included abdominal Ultrasound and MRI, chest CT, echocardiography, electrocardiography, and electroencephalography (EEG).

### Genetic analysis

Genomic DNA was extracted from peripheral blood samples using the QIAamp DNA Mini Kit (Qiagen, Germantown, MD, USA) per manufacturer’s directions. DNA quality and concentration were assessed by agarose gel electrophoresis and spectrophotometry (A260 nm). Whole-exome sequencing (WES) was performed according to a CAP/CLIA validated standard operating protocol. In brief, exome capture was conducted using the xGen Exome Research Panel v2 (Integrated DNA Technologies, Coralville, IA, USA), and sequencing was executed on the NovaSeq 6000 platform (Illumina, San Diego, CA, USA) to generate 150 bp paired-end reads. Raw sequencing data were aligned to the Genome Reference Consortium Human Build 37 (GRCh37) and Revised Cambridge Reference Sequence (rCRS) of the mitochondrial genome. The captured region comprised 34,366,188 bases, which is nearly 99.3% of the RefSeq protein coding region. Approximately 99.10% of the targeted bases were covered to a depth of ≥ 20x. The alignment was conducted using BWA-MEM, and variant calling was carried out using GATK v.3 [39, 40]. Copy number variants were analyzed using CoNIFER v0.2.2 and 3bCNV, an internally designed tool that utilizes exon-level depth-of-coverage information [41].

Variants were annotated using the Ensembl Variant Effect Predictor (VEP) and subsequently filtered and prioritized using EVIDENCE, an automated variant interpretation pipeline developed by 3 billion Laboratory (Seoul, South Korea) [42]. The EVIDENCE system integrates multiple computational tools and databases to automatically prioritize genetic variants based on: (1) the biological function of each gene; (2) the molecular, structural, and physicochemical impact of each variant predicted by in silico tools; (3) population frequency from gnomAD; (4) phenotype-gene relationship matching using Human Phenotype Ontology (HPO) terms; and (5) guidelines recommended by the American College of Medical Genetics and Genomics (ACMG) and the Association for Molecular Pathology (AMP) [42, 43]. The primary filtering criterion applied was the BA1 rule (stand-alone benign), which excludes common variants with minor allele frequency > 5% in population databases. Rare variants known to be associated with human disease were retained for analysis. No additional variant allele frequency cut-offs or hard filtering criteria were applied beyond the GATK Best Practices recommendations. After manual review by medical geneticists and physicians, variants deemed most likely to account for the patient’s phenotype were selected for clinical reporting. Based on internal validation studies demonstrating high accuracy of the variant calling pipeline, Sanger sequencing was reserved for variants with low confidence sequencing metrics and was therefore not performed for the high quality variants reported in this study.

Identified variants were described according to Human Genome Variation Society (HGVS) Nomenclature (v.21.1.1; https://hgvs-nomenclature.org) [44] using NCBI Reference Sequences for *TSC1* (NG_012386.1; NM_000368.5; NP_000359.1) and *TSC2* (NG_005895.1; NM_000548.5; NP_000539.2) and were validated using Mutalyzer v3.1.1 (https://mutalyzer.nl/). Variants were cross-checked with publicly available databases, including Genome Aggregation Database (gnomAD) v2.1.1 (https://gnomad.broadinstitute.org/), Leiden Open Variation Database (LOVD; https://www.lovd.nl/), ClinVar (https://www.ncbi.nlm.nih.gov/clinvar/), and Human Gene Mutation Database (HGMD; https://www.hgmd.cf.ac.uk/ac/index.php). Their functional effects were assessed using various bioinformatics tools, including Variant Effect Predictor (VEP; https://ensembl.org/Tools/VEP), PolyPhen-2 (http://genetics.bwh.harvard.edu/pph2/), MutationTaster2021 (https://www.mutationtaster.org/), Combined Annotation Dependent Depletion (CADD; https://cadd.gs.washington.edu/), Sorting Intolerant From Tolerant (SIFT; https://sift.bii.a-star.edu.sg/), MaxEntScan (http://hollywood.mit.edu/burgelab/maxent/Xmaxentscan_scoreseq.html), SpliceAI (https://spliceailookup.broadinstitute.org/), and Human Splicing Finder (HSF; https://hsf.genomnis.com/mutation/analysis). Functional domains were defined based on prior studies and UniProt annotations [14, 45, 46]. In brief, TSC1 consists of an N-terminal interaction domain (exons 4–8), a central tuberin-binding domain (exons 9–13), and a C-terminal coiled-coil motif (exons 17–23). TSC2 encompasses an N-terminal hamartin-binding (exons 1–22), a central region containing HEAT repeats (exons 23–33), and a C-terminal RHEB-specific GTPase-activating protein (RHEB-GAP) domain (exons 34–42).

### Data analysis

Data were analyzed using Stata/BE 17 (StataCorp LLC, College Station, TX, USA). Categorical variables were summarized as frequencies and percentages. Quantitative variables that were not normally distributed were presented as medians with interquartile ranges. Given the small sample size and marked heterogeneity of variants, no inferential or correlation analyses were attempted, as such comparisons would be statistically underpowered, prone to confounding, and potentially misleading. Therefore, the analysis was restricted to descriptive statistics, consistent with common practice in small rare-disease cohorts.

## Results

This study included 23 patients with TSC from 20 unrelated families. Table 1 summarizes the main characteristics of the study population. The demographic, disease onset, and genetic features are detailed in Table 2. The cohort included 16 males and 7 females, with a median age of 8.1 years (interquartile range [IQR] 4–15.8 years). Parental consanguinity was present in 13 cases (57%), and a positive family history of TSC was reported in 12 (52%). Patients initially presented with seizures (19/23, 83%) or developmental delay (4/23, 17%) at a median age of 8 months (IQR 3–24 months).

**Table 1:** Summary of main features in 23 patients with tuberous sclerosis complex

Feature	Number (%) / median (IQR)
Males/females	16/7
Current age (year)	8.1 (4–15.8)
Age at onset (month)	8 (3–24)
Presenting feature	
Seizures	19 (83%)
Developmental delay	4 (17%)
Neurological features	
Seizures	20 (87%)
TANDs	18 (78%)
ID	15 (65%)
DLD	15 (65%)
ASD	12 (52%)
Sleep difficulties	6 (26.1%)
Brain imaging	
Cortical tubers	22 (96%)
SENs	19 (83%)
SEGA	1 (4.4%)
Skin manifestations	
Hypomelanotic macules	23 (100%)
Facial angiofibroma	15 (65%)
Shagreen patch	11 (48%)
Ungual fibroma	8 (35%)
Oral fibroma	6 (26%)
Extra-neurocutaneous features	
Renal AMLs	10 (44%)
Renal cysts	6 (26%)
Cardiac rhabdomyoma	9 (39%)
Hepatic tumors	5 (22%)
Retinal hamartoma	4 (22%)^*^
Pulmonary LAM	0
Identified variants	
* TSC*	21 (91%)
* TSC1*	2 (7%)

*AML* Angiomyolipoma, *ASD* autism spectrum disorder, *DLD* delayed language development, *ID* intellectual disability, *LAM* Lymphangioleiomyomatosis, *SEGA* subependymal giant cell astrocytoma, *SENs* subependymal nodules, *TANDs* Tuberous sclerosis complex-associated neuropsychiatric disorders

^*^ Assessed in only 18 out of the 23 patients

**Table 2:** Demographic, age at onset, and genetic features of 23 patients with tuberous sclerosis complex

Patient ID^†^	Consan- guinity	Family history	Sex	Current age	Ag at onset	Presenting feature	Genetic variant^*^
1	–	+	F	4 y	3 mo	Seizures	*TSC2*: c.826_827del
2	–	–	M	13 y	2 mo	Seizures	*TSC2*: c.5238_5255del
3	–	+	F	15.8 y	18 mo	Seizures	*TSC2*: c.2537del
4 (3s)	–	+	F	5 y	2 y	Seizures	*TSC2*: c.2537del
5	–	–	M	7.3 y	3 mo	Seizures	*TSC2*: c.2599dup
6	+	–	M	7.8 y	1 mo	Seizures	*TSC2*: c.976-15G > A
7	–	–	F	3 y	4 mo	Seizures	*TSC2*: c.3385dup
8	+	–	M	19 y	8 mo	Seizures	*TSC2*: c.775–2 A > G
9	+	+	M	12.5 y	3 y	Delayed development	*TSC2*: c.976-14G > A
10 (9b)	+	+	M	17.1 y	3 y	Delayed development	*TSC2*: c.976-14G > A
11 (9b)	+	+	M	24.7 y	2 y	Delayed development	*TSC2*: c.976-14G > A
12	+	–	F	15.7 y	6 mo	Seizures	*TSC2*: c.3206_3207del
13	+	+	M	8.7 y	2 y	Delayed development	*TSC2*: c.600–2 A > G
14	+	–	M	3.4 y	3 mo	Seizures	*TSC2*: c.1067_1068dup
15	+	–	M	23.3 y	15 d	Seizures	*TSC2*: c.225 + 1G > A
16	–	–	F	17.7 y	2 y	Seizures	*TSC2*: c.2098G > A
17	–	+	M	8.1 y	3 y	Seizures	*TSC2*: c.5280del
18	+	+	F	2.5 y	8 mo	Seizures	*TSC2*: c.5238_5255del
19	+	+	M	5 y	2 y	Seizures	*TSC2*: c.5280del
20	–	+	M	15.1 y	6 mo	Seizures	*TSC2*: c.4258_4261del
21	+	–	M	2.4 y	4 mo	Seizures	*TSC2*: c.1659 C > A
22	–	–	M	7.5 y	2 y	Seizures	*TSC1*: c.1798 C > T
23	+	+	M	3.6 y	2 y	Seizures	*TSC1*: c.1525 C > T

^*^ Variants are described according to the Human Genome Variation Society Nomenclature v.21.1.1 (https://hgvs-nomenclature.org) using NCBI Reference Sequences: *TSC2*: NM_000548.5; *TSC1*: NM_000368.5

^**†**^ Sib pairs are indicated as b (brother) and s (sister)

–, absent; +, present; F, female; M, male; mo, month; y, year

The clinical and imaging features of individual patients are described in Table 3, and detailed seizure characteristics are provided in Additional file 2. Seizures were observed in 20 of the 23 patients (87%), with a median age at onset of 7 months (IQR 3–24 months). Focal seizures were the most common, affecting 18 patients (90%), followed by epileptic spasms in 12 (60%) and generalized seizures, either myoclonic or tonic–clonic, in 8 (40%); most patients (17/20; 85%) experienced more than one seizure type. On EEG, hypsarrhythmia was observed in 55% of patients, focal or multifocal epileptiform discharges in 95%, and generalized spike–wave or polyspike discharges in 30%. All patients required multiple antiseizure medications (ASMs). Vigabatrin (60%) and adrenocorticotropic hormone (40%) were used most often for epileptic spasms, and levetiracetam (95%) was the most frequent adjunctive agent. Other ASMs included oxcarbazepine (45%), sodium valproate (30%), topiramate (25%), and clonazepam (20%). Overall, 8 patients (40%) had drug-resistant epilepsy, while the remaining 12 (60%) demonstrated a partial response to ASMs.

**Table 3:** Clinical and imaging features of 23 patients with tuberous sclerosis complex

Patient ID	Seizure	TANDs				Hypome-lanotic macules	Facial ang-iofibroma	Shagreen patch	Ungual fibroma	Oral fibroma	Brain imaging			Retinal hamar-toma	Renal AML	Hepatic tumor/ cyst	Cardiac rhabdo-myoma	Pulmonary LAM
		ID	DLD	ASD	Sleep problems						Brain tubers	SENs	SEGA					
1	+	–	+	–	–	+	+	–	–	–	+	+	–	–	–	–	+	–
2	+	+	+	+	–	+	–	–	–	–	+	–	–	+	+	–	+	–
3	+	+	+	+	+	+	+	+	+	–	+	+	–	+	+	+	–	–
4	+	–	+	–	–	+	–	–	–	–	+	+	–	–	–	–	–	–
5	+	–	+	–	–	+	+	+	–	–	+	+	–	+	–	–	+	–
6	+	+	–	+	–	+	+	–	–	–	+	–	–	–	–	–	+	–
7	+	+	+	+	–	+	–	–	–	–	+	+	–	–	–	–	+	–
8	+	+	+	+	+	+	+	+	+	+	+	+	–	NA	+	–	–	–
9	–	+	–	+	–	+	+	+	+	+	+	+	–	–	+	+	–	–
10	–	+	–	+	–	+	+	+	+	+	+	+	–	–	+	+	–	–
11	+	+	+	+	–	+	+	+	+	+	+	+	–	–	+	–	–	–
12	+	+	+	+	+	+	+	+	+	+	+	+	–	NA	+	–	–	–
13	–	+	+	+	+	+	+	–	–	–	+	+	–	–	–	–	–	–
14	+	+	+	–	–	+	–	–	–	–	+	+	–	–	–	–	+	–
15	+	+	+	+	+	+	+	+	+	–	+	–	+	+	+	+	–	–
16	+	–	–	–	–	+	+	+	–	–	+	+	–	NA	+	–	–	–
17	+	–	–	–	–	+	+	–	–	–	+	+	–	NA	–	–	–	–
18	+	+	+	–	–	+	–	–	–	–	+	+	–	NA	–	–	+	–
19	+	–	–	–	–	+	–	–	–	–	+	+	–	–	–	–	–	–
20	+	+	+	+	+	+	+	+	+	+	+	+	–	–	+	+	–	–
21	+	+	+	–	–	+	–	–	–	–	–	–	–	–	–	–	+	–
22	+	–	–	–	–	+	–	+	–	–	+	+	–	–	–	–	–	–
23	+	–	–	–	–	+	+	–	–	–	+	+	–	–	–	–	+	–

–, absent; +, present; AML, angiomyolipoma; ASD, autism-spectrum disorder; DLD, delayed language development; ID, intellectual disability; F, female; LAM, lymphangioleiomyomatosis, M, male; mo, month; NA, not applicable; SEGA, subependymal giant cell astrocytoma; SEN, subependymal nodules; TANDs, tuberous sclerosis complex-associated neuropsychiatric disorders; y, year

**Fig. 1:**
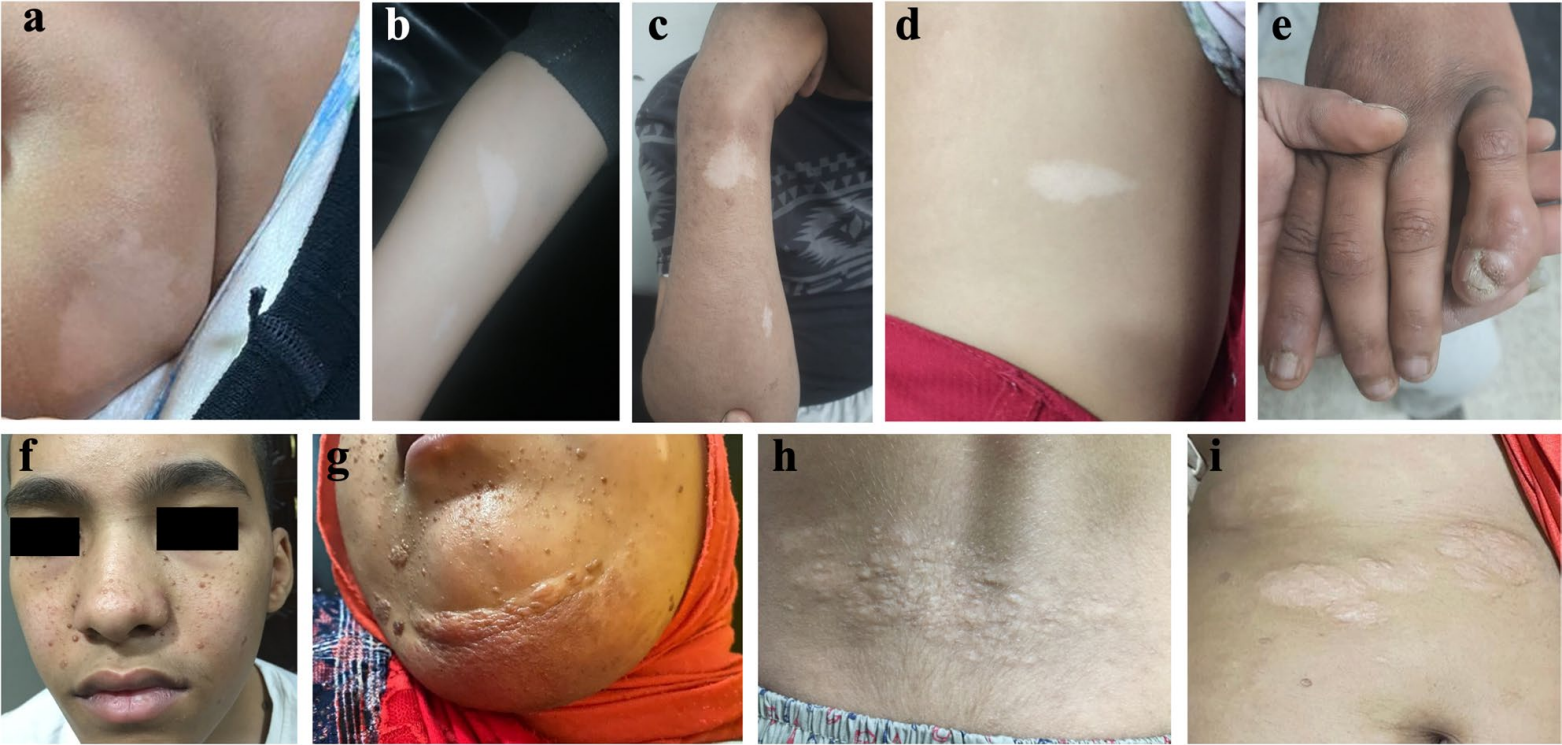
Cutaneous manifestations in patients with tuberous sclerosis complex. Hypomelanotic macules on buttocks (**a**), arms (**b**) and (**c**), and chest (**d**); ungual fibroma (**e**), facial angiofibromas (**F**) and (**g**); fibrous cephalic plaques (**g**); and shagreen patches on back (**h**) and abdomen (**i**)

**Fig. 2:**
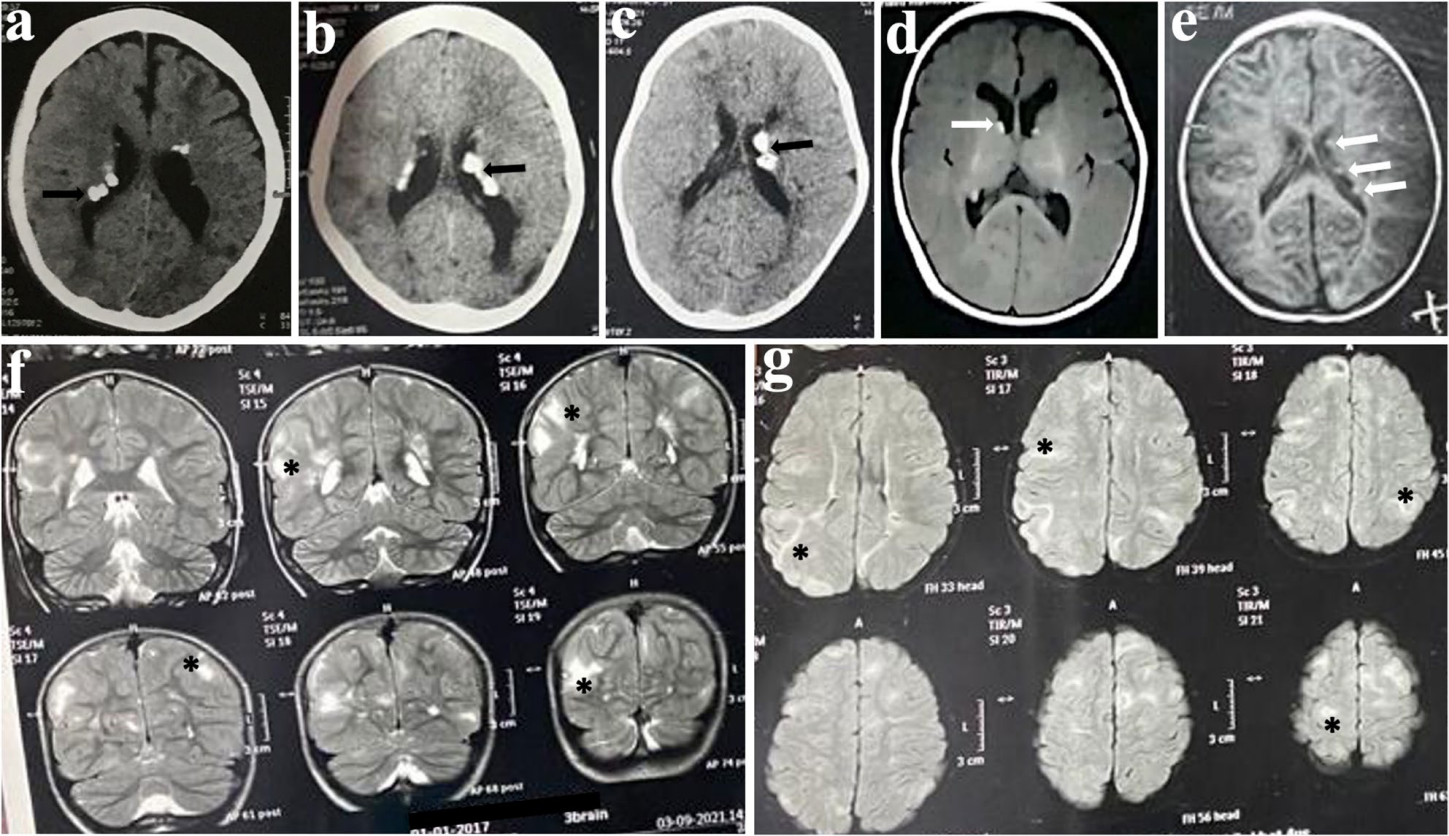
Representative brain imaging findings in patients with tuberous sclerosis complex. Brain axial CT (**a** – **c**), axial T1WI MRI (**d**, **e**), coronal T2WI (**f**), and axial GRE WI (**g**) sequences. Multiple subependymal nodules are observed along the lateral ventricular walls, particularly near the caudothalamic grooves, appearing as hyperdense lesions on axial CT images (**a – c**; black arrows) and isointense to hypointense on axial T1WI (**d** & **e**, white arrows). There are multiple cortical and subcortical tubers, appearing as hyperintense lesions (black asterisks) on coronal T2WI (**f**) and GRE WI (**g**) images with no blood signal

TANDs were identified in 18 patients (78%), including intellectual disability (ID) and delayed language development (each in 15 patients, 65%), autism spectrum disorder (ASD; 12 patients, 52%), and sleep difficulties (6 patients, 26.1%). Brain imaging revealed cortical tubers in 22 patients (96%), SENs in 19 (83%), and SEGA in one case (4%). All patients exhibited hypomelanotic macules. Other cutaneous findings included facial angiofibromas (15/23, 65%), shagreen patches (11/23, 48%), ungual fibromas (8/23, 35%), and oral fibromas (6/23, 26%). Extra-neurocutaneous manifestations were renal AMLs (10/23, 44%) and cysts (6/23, 26%), cardiac rhabdomyomas (9/23, 39%), hepatic tumors (5/23, 22%), retinal hamartomas (4/18, 22%), and bone and dental anomalies (each in 2/18, 11%). Representative dermatological and brain imaging features are depicted in Figs. 1 and 2, respectively.

A descriptive comparison between patients with *TSC2* and *TSC1* variants is provided in Additional file 3. The two patients with *TSC1* variants presented at a later age than those with *TSC2* variants (median age: 2 years vs. 8 months) and did not exhibit TANDs, renal involvement, hepatic tumors, shagreen patches, ungual fibromas, or oral fibromas.

As detailed in Table 4, a total of 18 distinct variants were identified: 16 in *TSC2* and 2 in *TSC1*. These included nine deletions/insertions, five splice-site, three nonsense, and one missense variant. Most variants (16/18, 89%) were “private”, each occurring in a single family; only *TSC2* c.5238_5255del and c.5280del variants were detected in two families. Deletion/insertion variants were predicted to cause a shift of the reading frame, except *TSC2* c.5238_5255del, which was expected to result in an in-frame deletion (p.His1746_Arg1751del) that affects the RHEB-GAP domain. Among the splice-site variants in *TSC2*, c.225 + 1G > A was predicted to alter a canonical splice donor site, two (c.600–2 A > G and c.775–2 A > G) to disrupt canonical splice acceptor sites, and two (c.976-15G > A and c.976-14G > A) to affect the polypyrimidine tract adjacent to the splice acceptor sites. The majority of variants (16/18) were classified as pathogenic or likely pathogenic, whereas two *TSC2* variants (c.2098G > A and c.5280del) were classified as variants of uncertain significance (VUS). The *TSC2* c.2098G > A (p.Glu700Lys) affects the hamartin-binding domain and was predicted to be “probably damaging” by PolyPhen-2 (score: 0.994), “damaging” by SIFT (score: 0.0), and “deleterious” by MutationTaster, and “moderate pathogenic” by CADD (score: 31). This variant was also predicted to affect splicing (MaxEntScan score reduced from 9.82 to 0.00; SpliceAI score: 0.39), with a likely intron retention of 40 nucleotides adjacent to exon 20 (Additional file 4). *TSC2* c.5280del was predicted to cause a frameshift leading to a truncated protein (p.Ser1761ProfsTer65); however, given its location near the 3′ end of the transcript, it is unlikely to undergo nonsense-mediated decay (NMD).

**Table 4:** Genetic variants identified in 23 Egyptian patients with tuberous sclerosis complex

Gene^*^	Nucleotide change	Predicted effect	I/E location	ACMG classification	gnomAD allele freq.	ClinVar accession	Reference
*TSC2*	c.225 + 1G > A	Aberrant splicing (exon 4 skipping)	I03	Pathogenic	NF	VCV000626196.11	This study
*TSC2*	c.600–2 A > G	Aberrant splicing (exon 7 skipping)	I06	Likely pathogenic	NF	VCV000049903.2	[11, 22]
*TSC2*	c.775–2 A > G	Aberrant splicing (exon 9 skipping)	I08	Likely pathogenic	NF	VCV001705534.4	This study
*TSC2*	c.826_827del	p.Met276ValfsTer61	E09	Pathogenic	NF	VCV000049447.41	[11, 22, 47]
*TSC2*	c.976-15G > A	Aberrant splicing (cryptic splice site in exon 11; exon 11 skipping)	I10	Pathogenic	NF	VCV000049396.26	[11, 25, 48–50]
*TSC2*	c.976-14G > A	Aberrant splicing (exon 11 skipping)	I10	Likely pathogenic	NF	VCV000065256.2	[51]
*TSC2*	c.1067_1068dup	p.Ala357TrpfsTer7	E11	Likely pathogenic	NF	VCV001705434.1	This study
*TSC2*	c.1659 C > A	p.Tyr553Ter	E16	Likely pathogenic	NF	VCV002444425.1	This study
*TSC2*	c.2098G > A	p.Glu700Lys/aberrant splicing	E20	VUS	NF	VCV000049732.3	[52]
*TSC2*	c.2537del	p.Phe846SerfsTer48	E22	Likely pathogenic	NF	VCV001705633.1	This study
*TSC2*	c.2599dup	p.Ser867LysfsTer16	E23	likely pathogenic	NF	VCV001705287.1	This study
*TSC2*	c.3206_3207del	p.Val1069AspfsTer98	E28	Pathogenic	NF	VCV000049246.30	[53, 54]
*TSC2*	c.3385dup	p.Arg1129ProfsTer39	E29	Likely pathogenic	NF	VCV001705626.1	This study
*TSC2*	c.4258_4261del	p.Ser1420GlyfsTer55	E34	Pathogenic	NF	VCV000050083.11	[20, 22, 55]
*TSC2*	c.5238_5255del	p.His1746_Arg1751del	E41	Pathogenic	NF	VCV000012402.60	[11, 15, 22, 23, 25]
*TSC2*	c.5280del	p.Ser1761ProfsTer65	E42	VUS	NF	VCV002444080.1	This study
*TSC1*	c.1525 C > T	p.Arg509Ter	E15	Pathogenic	NF	VCV000048796.49	[21, 56]
*TSC1*	c.1798 C > T	p.Gln600Ter	E15	Likely pathogenic	NF	VCV001705495.1	This study

*ACMG* American College of Medical Genetics and Genomics, *E* exon, *I* intron, *NF* not found, *VUS* variant of uncertain significance

*Variants are described according to the Human Genome Variation Society Nomenclature v.21.1.1 (https://hgvs-nomenclature.org) using the following NCBI Reference Sequences: *TSC2*: NM_000548.5, NP_000539.2; and *TSC1*: NM_000368.5 and NP_000359.1

Nine novel variants were identified in this cohort: *TSC1* c.1798 C > T (p.Gln600Ter) and eight in *TSC2*: c.225 + 1G > A, c.775–2 A > G, c.1067_1068dup (p.Ala357TrpfsTer7), c.1659 C > A (p.Tyr553Ter), c.2537del (p.Phe846SerfsTer48), c.2599dup (p.Ser867LysfsTer16), c.3385dup (p.Arg1129ProfsTer39), and c.5280del (p.Ser1761ProfsTer65). Of these, seven were truncating variants, six of which were expected to result in NMD. The c.225 + 1G > A was predicted to disrupt the canonical splice donor site of intron 3, leading to exon 4 skipping, while c.775–2 A > G was predicted to impact the canonical splice acceptor site of intron 8, likely resulting in exon 9 skipping.

Notably, Case 23 also harbored a pathogenic heterozygous variant in *LDLR* (NM_000527.5:c.542 C > T; NP_000518.1:p.Pro181Leu), associated with familial hypercholesterolemia type 1 (MIM **#**143890). This patient exhibited elevated serum levels of total cholesterol (254 mg/dl) and low-density lipoprotein cholesterol (183 mg/dL). No other remarkable laboratory abnormalities were observed in this or any of the remaining cases. Importantly, no additional candidate variants in genes other than *TSC1* and *TSC2* were identified at the time of analysis that could explain the observed TSC phenotypes.

All patients received multidisciplinary care and surveillance in accordance with the 2021 updated international TSC surveillance and management recommendations [1]. Importantly, Case 15, who had SEGA, was the only patient treated with mTOR inhibitors and who also underwent surgical intervention.

## Discussion

This study represents the first comprehensive genetic analysis of TSC among Egyptian patients. In our cohort of 23 patients, we identified 18 distinct variants, nine of which were novel, with a predominance of *TSC2* variants (16/18) and a high proportion of “private” variants, emphasizing the marked genetic heterogeneity of TSC in this population. The observed phenotypic features largely aligned with previous reports, though some differences were noted. The findings broaden the demographic, phenotypic, and molecular spectrum of TSC by identifying nine novel variants and highlighting distinct regional trends–such as a higher proportion of familial cases, a predominance of *TSC2* variants (89%), and higher frequencies of TANDs and cortical tubers–in an underrepresented population.

The distribution of TSC manifestations in the present cohort broadly aligns with data from other studies, although certain variations are evident, often due to differences in age and diagnostic approaches [11, 12, 15–17, 19, 21, 23, 26–29, 57, 58]. Compared with baseline findings from the TuberOus SClerosis registry to increase disease Awareness (TOSCA) registry (the largest international TSC cohort) [19], the frequencies of our CNS features were similar for seizures (87% vs. TOSCA: 83.5%) and SENs (83% vs. 78.2%), higher for TANDs (78.3% vs. 57.8%) and cortical tubers (96% vs. 82.2%), and lower for SEGA (4.4% vs. 24.4%). Compared with a recently published TSC cohort (81 patients) from Sicily [58], our study showed frequencies that are higher for seizures (87% vs. 69%), TANDs (ID: 65% vs. 54.3%; ASD 52% vs. 12.4%; sleep abnormalities 26.1% vs. 6.2%), and cortical tubers (96% vs. 87.7%), similar for SENs (83% vs. 80.3%), and lower for SEGA (4.4% vs. 11%). Among patients with seizures, 40% were drug-resistant, in agreement with previously reported percentages of 50–65% [16, 19, 59–61]. Longitudinal studies have demonstrated that early-onset, severe, and prolonged seizures are associated with a higher risk of TANDs and ID [62, 63]. The elevated frequencies of TANDs and cortical tubers in our cohort may reflect more systematic neuropsychiatric and imaging evaluations compared to the TOSCA study, which included patients from multiple countries—some with less developed health care systems—and reported high percentages of missing data (up to 86% in certain domains), suggesting underdiagnosis [19]. At the same time, recruitment of cases from tertiary neurology clinics in our study may have introduced ascertainment bias, resulting in overrepresentation of individuals with more pronounced neurological involvement. Taken together, both underdiagnosis in large multicenter registries and selective recruitment in our cohort may collectively explain the observed differences. Another contributing factor could be the higher frequency of *TSC2* variants in our study (88.9%) compared with the TOSCA (76.2%) and Sicilian (61.7%) cohorts [19, 58], as *TSC2* variants are generally associated with a more severe neurological phenotype [5, 6, 14, 58]. Conversely, the relatively low frequency of SEGA in our cohort may be attributed to differences in imaging follow-up and diagnostic definitions, since SEGA often develops gradually and lacks a consensus definition. These factors likely contribute to the wide variation in SEGA prevalence (5% − 30%) reported across different TSC cohorts [11, 16, 26, 61, 64, 65].

Skin manifestations, such as hypomelanotic macules (100%), facial angiofibromas (65%), shagreen patches (48%), and ungual fibromas (35%), generally occurred at frequencies similar to those described in prior studies [11, 15, 16, 18, 21, 26, 66]. The frequencies of renal AMLs (44%) and cysts (26%), retinal hamartomas (22%), and hepatic tumors (22%) were also within previously reported ranges [11, 15, 18–21, 25–27, 57, 59, 66]. However, cardiac rhabdomyomas were detected in only 39% of patients, which is lower than the 45–97% reported in other cohorts [10, 11, 15, 20, 61, 67, 68], and no pulmonary abnormalities were observed. These discrepancies may be explained by the age-dependent evolution of certain TSC manifestations [3, 14]. For instance, cardiac rhabdomyomas develop *in utero* and typically regress after birth and thus may be missed unless identified antenatally or during early infancy. In contrast, pulmonary and renal manifestations often emerge later and increase in frequency with age [13, 61, 68]. Therefore, in our cohort with a median age of 8 years, some patients may have previously had cardiac rhabdomyomas that regressed before time at diagnosis, while others not exhibiting kidney or lung manifestations at the time of evaluation may develop these features over time.

*TSC2* variants were more common than *TSC1* variants in our study, which is consistent with findings from diverse populations, including British [12], Danish [22, 24], Dutch [26], Sicilians [58], Japanese [29], Chinese [16, 21], Taiwanese [18], Korean [20, 27], Indian [28], Saudi [59], American [11, 15], Mexican [23], and Brazilian [17, 25] cohorts. Similarly, the only prior molecular study from Egypt identified two pathogenic *TSC2* variants (c.4375 C > T and c.1513 C > T) in three of five infants with large cardiac rhabdomyomas [30]. The higher frequency of *TSC2* variants may be attributed to the gene’s larger coding region and greater number of exons, increasing the likelihood of certain variants, such as missense, splice site, and genomic rearrangement [5, 11, 26]. Interestingly, the *TSC2*/*TSC1* variant ratio in our study (8:1) is higher than those reported in the TOSCA (3.2:1) [19] and Sicilian (1.6:1) [58] studies, despite a high percentage of familial cases (52%), in whom *TSC1* variants are typically more common (~ 50%) than in sporadic cases [12, 14, 15]. This unusually high proportion of familial cases, compared with the typically reported one-third proportion [5, 11, 12, 15, 16, 18, 19, 21–23, 26, 28, 66], may be explained by the high percentage of consanguinity in our cohort (~ 57%). Although TSC is an autosomal dominant disease whose overall population frequency is not directly influenced by consanguinity, high consanguinity likely facilitates greater clustering and recognition of familial cases, particularly in extended families sharing a common genetic background [69, 70].

Most variants identified in the present cohort (16/18) were “private”, consistent with the known genetic heterogeneity of TSC [4, 5, 7]. This predominance of “private” variants underscores the need for individualized genetic counseling, as recurrence risk estimation is often challenging in the absence of recurrent or founder variants [1, 4, 6, 48].

Three patients (Cases 16, 17, and 19; 13% of the cohort) met definite clinical diagnostic criteria for TSC but had no pathogenic or likely pathogenic variants identified in *TSC1* or *TSC2* by WES. However, Cases 17 and 19 both harbor the same VUS (*TSC2* c.5280del), while Case 16 carries *TSC2* c.2098G > A, also classified as VUS. Both variants are strong candidates for pathogenicity based on their predicted effects and compatibility with the TSC phenotype. The *TSC2* c.5280del variant is predicted to cause a frameshift (p.Ser1761ProfsTer65); however, its location in exon 41 (out of total 42 exons) raises uncertainty about whether it undergoes NMD or produces a C-terminally truncated protein lacking part of the RHEB-GAP domain. The *TSC2* c.2098G > A variant was predicted to cause not only a direct protein damaging effect (p.Glu700Lys) but also aberrant splicing. The current VUS classifications reflect these mechanistic uncertainties and absence of functional validation, rather than evidence against pathogenicity. With functional studies (e.g., minigene splicing assays, Western blot) and additional segregation data, these variants could be reclassified as likely pathogenic or pathogenic [42, 43], which could help clarify the molecular diagnosis in these unresolved cases. Indeed, all these three patients exhibited classical TSC manifestations including hypomelanotic macules, seizures, cortical tubers, and SENs, fully consistent with definite TSC [1].

Considering only pathogenic and likely pathogenic variants, the diagnostic yield in our study (20/23; 87%) is comparable to rates (~ 85%) reported in large international cohorts [6, 11, 12]. Several mechanisms may account for the absence of definitive molecular diagnosis, including low-level mosaicism below the detection threshold of WES, deep intronic variants not captured by exome sequencing, or structural rearrangements missed by CNV algorithms [28, 48, 71]. For Cases 16, 17, and 19, additional investigations could include functional validation studies to support VUS reclassification; extended segregation analysis; deep targeted sequencing in multiple tissues; high-sensitivity parental testing; and whole-genome sequencing [1, 4, 6, 48]. These approaches would likely achieve near-complete molecular diagnosis.

A large proportion of variants were truncating, including frameshift indels and nonsense, which agree with prior studies [3, 12, 14–18, 21, 23, 27]. The variants were distributed across multiple exons and introns, with no obvious mutational hotspots. Indeed, some recurrently affected exons have been infrequently described, such as exons 8 and 15 in *TSC1* [14, 16, 21, 23, 27, 66] and exons 16, 34, and 41 in *TSC2* [12, 14, 16, 27, 66]. No large genomic rearrangements were detected, although such events are known to occur in up to 1% of *TSC1* and 10% of *TSC2* cases [28, 71].

Although formal genotype-phenotype correlations were not feasible due to the small sample size and remarkable variant diversity, it is worth noting that the two patients with *TSC1* variants presented later than those with *TSC2* variants (median age: 2 years vs. 8 months) and lacked TANDs, renal affection, or hepatic tumors. Previous genotype-phenotype studies have shown that *TSC2* variants are associated with more severe phenotypes, including earlier seizure onset, drug-resistant epilepsy, ID, and involvement of other organs [5, 6, 11–15, 20, 21, 26, 29, 58, 68, 71]. This may stem from two possible mechanisms: (1) the larger size of *TSC2* makes it more susceptible to second-hit events (somatic inactivation of the second allele as per the Knudson two-hit model) [7, 13], and (2) tuberin dysfunction more directly disrupts mTOR regulation than hamartin loss, which mainly affects complex stability [11, 13, 65]. Notably, the sibling pairs in our cohort shared a similar disease spectrum but not identical phenotypes. This observation is consistent with prior reports describing intrafamilial variability in TSC, in which relatives or even monozygotic twins carrying the same variant may exhibit divergent neurological and systemic features [4, 6, 72, 73]. Such variable expressivity may be attributable to second-hit effects, modifier genes, or environmental influences [4, 6, 72, 73].

While genotype alone does not fully predict clinical severity in TSC, early genetic diagnosis remains essential. It enables confirmation of TSC in patients not yet fulfilling clinical diagnostic criteria, allows for early management and surveillance, and informs genetic counseling and reproductive planning [3, 7]. Moreover, emerging evidence supports the use of presymptomatic interventions, such as vigabatrin and mTOR inhibitors, to mitigate neurological injury and improve outcomes [7, 64]. For instance, the EpiSTOP trial demonstrated that preventive vigabatrin therapy in young infants with early interictal epileptiform discharges delayed the onset of clinical seizures and reduced the risk of drug-resistant epilepsy, though neurodevelopmental outcomes were unchanged at 2 years of age [74].

Despite the demonstrated efficacy of mTOR inhibitors in managing various TSC-related manifestations (e.g., intractable seizures, large SEGA, renal AML, and pulmonary LAM) [1, 2, 60], only one patient in our cohort received these agents, primarily due to logistical and financial constraints. Similarly, the frequent use of brain CT in the initial diagnostic work-up was often driven by limited access to MRI. These findings highlight the persistent challenges faced by individuals with rare diseases like TSC in resource-limited settings [75, 76]. Addressing these gaps requires strengthening national rare disease frameworks, ensuring sustainable drug procurement, and incorporating mTOR inhibitors into essential medicines lists or insurance coverage schemes. Establishing multidisciplinary TSC clinics and regional registries could further promote equitable access to advanced diagnostics and targeted therapies [75, 76].

The present study has some limitations. First, the recruitment from tertiary neurology clinics might have introduced ascertainment bias, potentially overrepresenting patients with more severe phenotypes. Moreover, the WES approach used standard GATK variant calling pipelines, which are optimized for germline variant detection and may not reliably identify low-level somatic mosaicism. Mosaic variants in *TSC1* or *TSC2* have been reported in up to 10–15% of TSC cases and may require tissue-specific analysis or deep targeted sequencing for detection [48]. Additionally, the small sample size and the high heterogeneity of identified variants (16 unique *TSC2* variants and only 2 *TSC1* variants) precluded robust statistical comparisons, such as genotype-phenotype correlations, because of low statistical power and the potential for confounding or misleading conclusions. Finally, the present study did not include functional validation assays (e.g., cDNA or RNA sequencing, minigene assays, or Western blotting) or extended segregation analyses. Incorporating these approaches in future research could help clarify the pathogenicity of the two VUS (*TSC2* c.2098G > A and c.5280del) and support their potential reclassification according to ACMG guidelines. Although EVIDENCE performs comprehensive variant prioritization, some VUS may be reclassified in the future as additional functional or segregation evidence becomes available; however, except *LDLR* c.542 C > T, no other candidate variants in genes other than *TSC1* and *TSC2* were identified at the time of analysis.

## Conclusion

This study presents the first comprehensive genetic analysis of TSC in Egyptian patients. The phenotypic findings were largely consistent with previous reports, although certain features—such as SEGA, cardiac rhabdomyoma, and pulmonary involvement—were less frequent, likely reflecting the age distribution and diagnostic context of our cohort. Eighteen distinct variants were detected, nine of which are novel, with a predominance of *TSC2* variants (16/18). Most variants were “private”, highlighting the genetic heterogeneity of TSC. Collectively, these findings broaden the demographic, phenotypic, and molecular spectrum of TSC and provide important data from an underrepresented population.

## Supplementary Information

Additional file 1: STROBE checklist.

Additional file 2: Characteristics of seizures in 20 patients with tuberous sclerosis complex.

Additional file 3: Comparison between patients with *TSC2* and *TSC1* variants.

Additional file 4: Novel in silico prediction of splicing mechanism for TSC2 c.2098G>A variant identified in Case 16.

## Data Availability

All data generated during this study are included in this published article and its supplementary information files.

## Abbreviations

ACMG: American College of Medical Genetics and Genomics
AML: Angiomyolipomas
ASD: Autism spectrum disorder
ASMs: Antiseizure medications
CNS: Central nervous system
CT: Computed tomography
EEG: Electroencephalogram
ID: Intellectual disability
LAM: Lymphangioleiomyomatosis
MENA: Middle East North Africa
MRI: Magnetic resonance imaging
mTOR: Mechanistic target of rapamycin
NMD: Nonsense-mediated decay
RHEB-GAP: RHEB-specific GTPase-activating protein
SEGA: Subependymal giant cell astrocytoma
SENs: Subependymal nodules
TANDs: Tuberous sclerosis complex-associated neuropsychiatric disorders
TOSCA: TuberOus SClerosis registry to increase disease Awareness
TSC: Tuberous sclerosis complex
VUS: Variant of uncertain significance
WES: Whole exome sequencing

## References

[1] Northrup H, Aronow ME, Bebin EM, Bissler J, Darling TN, De Vries PJ, et al. Updated international tuberous sclerosis complex diagnostic criteria and surveillance and management recommendations. Pediatr Neurol. 2021;123:50–66. 10.1016/j.pediatrneurol.2021.07.011. 34399110 10.1016/j.pediatrneurol.2021.07.011

[2] Winden K, Bebin EM, Jeste S, Krueger DA, Paul E, Sahin M. Tuberous sclerosis complex. Nat Rev Dis Primer. 2026;12:11. 10.1038/s41572-026-00688-9. 10.1038/s41572-026-00688-9 41820375

[3] Marom D. Genetics of tuberous sclerosis complex: an update. Childs Nerv Syst. 2020;36:2489–96. 10.1007/s00381-020-04726-z. 32761379 10.1007/s00381-020-04726-z

[4] Peron A, Au KS, Northrup H. Genetics, genomics, and genotype–phenotype correlations of TSC: insights for clinical practice. Am J Med Genet C Semin Med Genet. 2018;178:281–90. 10.1002/ajmg.c.31651. 30255984 10.1002/ajmg.c.31651

[5] Tolliver S, Smith ZI, Silverberg N. The genetics and diagnosis of pediatric neurocutaneous disorders: neurofibromatosis and tuberous sclerosis complex. Clin Dermatol. 2022;40:374–82. 10.1016/j.clindermatol.2022.02.010. 35248688 10.1016/j.clindermatol.2022.02.010

[6] Curatolo P, Trivisano M, Specchio N. Updated genotype-phenotype correlations in TSC. Semin Pediatr Neurol. 2023;47:101086. 10.1016/j.spen.2023.101086. 37919037 10.1016/j.spen.2023.101086

[7] Northrup H, Koenig MK, Pearson DA, Au KS. Tuberous sclerosis complex. Seattle (WA): University of Washington, Seattle; 1999. 20301399

[8] Wang A, Wang C, Li W, Qiao J, Luo Y, Tian Y. Ocular manifestations in pediatric tumor suppressor gene mutations: a case series and literature review of RB1, NF1, NF2, VHL, and TSC. BMC Pediatr. 2025;25:371. 10.1186/s12887-025-05694-6. 40346602 10.1186/s12887-025-05694-6 PMC12065174

[9] Arredondo KH, Jülich K, Roach ES. Tuberous sclerosis complex: diagnostic features, surveillance, and therapeutic strategies. Semin Pediatr Neurol. 2024;51:101155. 10.1016/j.spen.2024.101155. 39389658 10.1016/j.spen.2024.101155

[10] Praticò AD, Lo Bianco M, Laganà A, Di Napoli C, Salafia S, Sciacca P, et al. Cardiac rhabdomyomas in tuberous sclerosis complex: clinical manifestations and genotype correlations. BMC Pediatr. 2025;26:36. 10.1186/s12887-025-06383-0. 41361250 10.1186/s12887-025-06383-0 PMC12817622

[11] Dabora SL, Jozwiak S, Franz DN, Roberts PS, Nieto A, Chung J, et al. Mutational analysis in a cohort of 224 tuberous sclerosis patients indicates increased severity of TSC2, compared with TSC1, disease in multiple organs. Am J Hum Genet. 2001;68:64–80. 10.1086/316951. 11112665 10.1086/316951 PMC1234935

[12] Jones AC, Shyamsundar MM, Thomas MW, Maynard J, Idziaszczyk S, Tomkins S, et al. Comprehensive mutation analysis of TSC1 and TSC2—and phenotypic correlations in 150 families with tuberous sclerosis. Am J Hum Genet. 1999;64:1305–15. 10.1086/302381. 10205261 10.1086/302381 PMC1377866

[13] Farach LS, Pearson DA, Woodhouse JP, Schraw JM, Sahin M, Krueger DA, et al. Tuberous sclerosis complex genotypes and developmental phenotype. Pediatr Neurol. 2019;96:58–63. 10.1016/j.pediatrneurol.2019.03.003. 31005478 10.1016/j.pediatrneurol.2019.03.003 PMC6837240

[14] Salussolia CL, Klonowska K, Kwiatkowski DJ, Sahin M. Genetic etiologies, diagnosis, and treatment of tuberous sclerosis complex. Annu Rev Genomics Hum Genet. 2019;20:217–40. 10.1146/annurev-genom-083118-015354. 31018109 10.1146/annurev-genom-083118-015354

[15] Au KS, Williams AT, Roach ES, Batchelor L, Sparagana SP, Delgado MR, et al. Genotype/phenotype correlation in 325 individuals referred for a diagnosis of tuberous sclerosis complex in the United States. Genet Med. 2007;9:88–100. 10.1097/gim.0b013e31803068c7. 17304050 10.1097/gim.0b013e31803068c7

[16] Ding Y, Wang J, Zhou S, Zhou Y, Zhang L, Yu L, et al. Genotype and phenotype analysis of chinese children with tuberous sclerosis complex: a pediatric cohort study. Front Genet. 2020;11:204. 10.3389/fgene.2020.00204. 32211034 10.3389/fgene.2020.00204 PMC7076134

[17] Dufner-Almeida LG, Cardozo LFM, Schwind MR, Carvalho D, Almeida JPG, Cappellano AM, et al. Molecular and functional assessment of TSC1 and TSC2 in individuals with tuberous sclerosis complex. Genes. 2024;15:1432. 10.3390/genes15111432. 39596632 10.3390/genes15111432 PMC11593644

[18] Hung C-C, Su Y-N, Chien S-C, Liou H-H, Chen C-C, Chen P-C, et al. Molecular and clinical analyses of 84 patients with tuberous sclerosis complex. BMC Med Genet. 2006;7:72. 10.1186/1471-2350-7-72. 16981987 10.1186/1471-2350-7-72 PMC1592085

[19] Kingswood JC, d’Augères GB, Belousova E, Ferreira JC, Carter T, Castellana R, et al. TuberOus SClerosis registry to increase disease awareness (TOSCA) – baseline data on 2093 patients. Orphanet J Rare Dis. 2017;12:2. 10.1186/s13023-016-0553-5. 28057044 10.1186/s13023-016-0553-5 PMC5217262

[20] Lee JS, Lim BC, Chae J, Hwang YS, Seong M, Park SS, et al. Mutational analysis of paediatric patients with tuberous sclerosis complex in Korea: genotype and epilepsy. Epileptic Disord. 2014;16:449–55. 10.1684/epd.2014.0712. 25498131 10.1684/epd.2014.0712

[21] Ng SYl, Luk H-M, EWl H, SSw C, KPt Y, Ho S, et al. Genotype/phenotype correlation in 123 Chinese patients with tuberous sclerosis complex. Eur J Med Genet. 2022;65:104573. 10.1016/j.ejmg.2022.104573. 35918040 10.1016/j.ejmg.2022.104573

[22] Rendtorff ND, Bjerregaard B, Frödin M, Kjaergaard S, Hove H, Skovby F, et al. Analysis of 65 tuberous sclerosis complex (TSC) patients by TSC2 < DGGE, TSC1/TSC2 MLPA, and TSC1 long-range PCR sequencing, and report of 28 novel mutations. Hum Mutat. 2005;26:374–83. 10.1002/humu.20227. 16114042 10.1002/humu.20227

[23] Reyna-Fabián ME, Hernández-Martínez NL, Alcántara-Ortigoza MA, Ayala-Sumuano JT, Enríquez-Flores S, Velázquez-Aragón JA, et al. First comprehensive TSC1/TSC2 mutational analysis in Mexican patients with tuberous sclerosis complex reveals numerous novel pathogenic variants. Sci Rep. 2020;10:6589. 10.1038/s41598-020-62759-5. 32313033 10.1038/s41598-020-62759-5 PMC7170856

[24] Rosengren T, Nanhoe S, De Almeida LGD, Schönewolf-Greulich B, Larsen LJ, Hey CAB, et al. Mutational analysis of TSC1 and TSC2 in Danish patients with tuberous sclerosis complex. Sci Rep. 2020;10:9909. 10.1038/s41598-020-66588-4. 32555378 10.1038/s41598-020-66588-4 PMC7303179

[25] Rosset C, Vairo F, Bandeira IC, Correia RL, De Goes FV, Da Silva RTB, et al. Molecular analysis of TSC1 and TSC2 genes and phenotypic correlations in Brazilian families with tuberous sclerosis. PLoS ONE. 2017;12:e0185713. 10.1371/journal.pone.0185713. 28968464 10.1371/journal.pone.0185713 PMC5624610

[26] Sancak O, Nellist M, Goedbloed M, Elfferich P, Wouters C, Maat-Kievit A, et al. Mutational analysis of the TSC1 and TSC2 genes in a diagnostic setting: genotype – phenotype correlations and comparison of diagnostic DNA techniques in tuberous sclerosis complex. Eur J Hum Genet. 2005;13:731–41. 10.1038/sj.ejhg.5201402. 15798777 10.1038/sj.ejhg.5201402

[27] Shin HJ, Lee S, Kim SH, Lee JS, Oh JY, Ko A, et al. Genotypic and phenotypic analysis of Korean patients with tuberous sclerosis complex. Neurogenetics. 2024;25:471–9. 10.1007/s10048-024-00777-5. 39110368 10.1007/s10048-024-00777-5

[28] Sudarshan S, Kumar A, Gupta A, Bhari N, Sethuraman G, Kaushal T, et al. Mutation Spectrum of tuberous sclerosis complex patients in Indian population. J Pediatr Genet. 2021;10:274–83. 10.1055/s-0040-1716495. 34849272 10.1055/s-0040-1716495 PMC8608467

[29] Togi S, Ura H, Hatanaka H, Niida Y. Genotype and phenotype landscape of 283 Japanese patients with tuberous sclerosis complex. Int J Mol Sci. 2022;23:11175. 10.3390/ijms231911175. 36232477 10.3390/ijms231911175 PMC9569560

[30] Al Kindi HN, Ibrahim AM, Roshdy M, Abdelghany BS, Yehia D, Masoud AN, et al. Clinical, cellular, and molecular characterisation of cardiac rhabdomyoma in tuberous sclerosis. Cardiol Young. 2021;31:1297–305. 10.1017/s1047951121000172. 33602381 10.1017/S1047951121000172

[31] Aladawy MA-A, Ismail AH, Mansour TMM, Ibrahim TA. Clinical and radiological assessment of children with tuberous sclerosis. Al-Azhar J Pediatr. 2025;28:4417–29. 10.21608/azjp.2025.420639.

[32] Megahed H, Hindawy AS, ElGwabi H. Targeted treatment of tuberous sclerosis complex in Egyptian children. Middle East J Med Genet. 2016;5:31–6. 10.1097/01.mxe.0000475218.07823.3c.

[33] Metwellay K, Farghaly H, Darweesh A, Hamed S. Cognitive delay in children with tuberous sclerosis in a developing country: clinical correlations. J Pediatr Epilepsy. 2015;01:221–8. 10.3233/pep-12034.

[34] Samir H, Ghaffar HA, Nasr M. Seizures and intellectual outcome: Clinico-radiological study of 30 Egyptian cases of tuberous sclerosis complex. Eur J Paediatr Neurol. 2011;15:131–7. 10.1016/j.ejpn.2010.07.010. 20817577 10.1016/j.ejpn.2010.07.010

[35] Shehata H, AbdelGhaffar H, Nasreldin M, Elmazny A, Abdelalim A, Sabbah A, et al. Clinical patterns and outcomes of status epilepticus in patients with tuberous sclerosis complex. Ther Clin Risk Manag. 2017;13:779–85. 10.2147/tcrm.s138576. 28721058 10.2147/TCRM.S138576 PMC5501639

[36] Beniczky S, Trinka E, Wirrell E, Abdulla F, Al Baradie R, Alonso Vanegas M, et al. Updated classification of epileptic seizures: position paper of the International League Against Epilepsy. Epilepsia. 2025;66:1804–23. 10.1111/epi.18338. 40264351 10.1111/epi.18338 PMC12169392

[37] De Vries PJ, Whittemore VH, Leclezio L, Byars AW, Dunn D, Ess KC, et al. Tuberous Sclerosis Associated Neuropsychiatric Disorders (TAND) and the TAND Checklist. Pediatr Neurol. 2015;52:25–35. 10.1016/j.pediatrneurol.2014.10.004. 25532776 10.1016/j.pediatrneurol.2014.10.004 PMC4427347

[38] American Psychiatric Association. editor. Diagnostic and statistical manual of mental disorders. 5th edition. Arlington, VA: American Psychiatric Association; 2013. 10.1176/appi.books.9780890425596.

[39] Li H, Durbin R. Fast and accurate short read alignment with Burrows–Wheeler transform. Bioinformatics. 2009;25:1754–60. 10.1093/bioinformatics/btp324. 19451168 10.1093/bioinformatics/btp324 PMC2705234

[40] Van Der Auwera GA, Carneiro MO, Hartl C, Poplin R, Del Angel G, Levy‐Moonshine A, et al. From FastQ data to high‐confidence variant calls: the genome analysis toolkit best practices pipeline. Curr Protoc Bioinforma. 2013;43:1.10.1-11.10.33. 10.1002/0471250953.bi1110s43. 10.1002/0471250953.bi1110s43 PMC4243306 25431634

[41] Krumm N, Sudmant PH, Ko A, O’Roak BJ, Malig M, Coe BP, et al. Copy number variation detection and genotyping from exome sequence data. Genome Res. 2012;22:1525–32. 10.1101/gr.138115.112. 22585873 10.1101/gr.138115.112 PMC3409265

[42] Seo GH, Kim T, Choi IH, Park J, Lee J, Kim S, et al. Diagnostic yield and clinical utility of whole exome sequencing using an automated variant prioritization system, EVIDENCE. Clin Genet. 2020;98:562–70. 10.1111/cge.13848. 32901917 10.1111/cge.13848 PMC7756481

[43] Richards S, Aziz N, Bale S, Bick D, Das S, Gastier-Foster J, et al. Standards and guidelines for the interpretation of sequence variants: a joint consensus recommendation of the American College of Medical Genetics and Genomics and the Association for Molecular Pathology. Genet Med. 2015;17:405–24. 10.1038/gim.2015.30. 25741868 10.1038/gim.2015.30 PMC4544753

[44] Den Dunnen JT, Dalgleish R, Maglott DR, Hart RK, Greenblatt MS, McGowan-Jordan J, et al. HGVS recommendations for the description of sequence variants: 2016 update. Hum Mutat. 2016;37:564–9. 10.1002/humu.22981. 26931183 10.1002/humu.22981

[45] UniProt Consortium. UniProt: the universal protein knowledgebase in 2023. Nucleic Acids Res. 2023;51:D523–31. 10.1093/nar/gkac1052. 36408920 10.1093/nar/gkac1052 PMC9825514

[46] Van Slegtenhorst M, Nellist M, Nagelkerken B, cheadle J, Snell R, Van Den Ouweland A, et al. Interaction between hamartin and tuberin, the TSC1 and TSC2 gene products. Hum Mol Genet. 1998;7:1053–7. 10.1093/hmg/7.6.1053. 9580671 10.1093/hmg/7.6.1053

[47] Niida Y, Lawrence-Smith N, Banwell A, Hammer E, Lewis J, Beauchamp RL, et al. Analysis of bothTSC1 andTSC2 for germline mutations in 126 unrelated patients with tuberous sclerosis. Hum Mutat. 1999;14(199911):412–22. 10.1002/(sici)1098-1004. )14:5%253C412::aid-humu7%253E3.0.co;2-k. 10533067 10.1002/(SICI)1098-1004(199911)14:5<412::AID-HUMU7>3.0.CO;2-K

[48] Tyburczy ME, Dies KA, Glass J, Camposano S, Chekaluk Y, Thorner AR, et al. Mosaic and intronic mutations in TSC1/TSC2 explain the majority of TSC patients with no mutation identified by conventional testing. PLOS Genet. 2015;11:e1005637. 10.1371/journal.pgen.1005637. 26540169 10.1371/journal.pgen.1005637 PMC4634999

[49] Mayer K, Ballhausen W, Leistner W, Rott H-D. Three novel types of splicing aberrations in the tuberous sclerosis TSC2 gene caused by mutations apart from splice consensus sequences. Biochim Biophys Acta BBA - Mol Basis Dis. 2000;1502:495–507. 10.1016/s0925-4439(00)00072-7. 10.1016/s0925-4439(00)00072-7 11068191

[50] Wang T, Wang J, Ma Y, Zhou H, Ding D, Li C, et al. High genetic burden in 163 Chinese children with status epilepticus. Seizure. 2021;84:40–6. 10.1016/j.seizure.2020.10.032. 33278787 10.1016/j.seizure.2020.10.032

[51] Blasco-Pérez L, Iranzo-Nuez L, López-Ortega R, Martínez-Cruz D, Camprodon-Gómez M, Tenés A, et al. An integral approach to the molecular diagnosis of tuberous sclerosis complex. J Mol Diagn. 2023;25:692–701. 10.1016/j.jmoldx.2023.06.006. 37356622 10.1016/j.jmoldx.2023.06.006

[52] Yamamoto T, Pipo JR, Feng J-H, Takeda H, Nanba E, Ninomiya H, et al. Novel TSC1 and TSC2 mutations in Japanese patients with tuberous sclerosis complex. Brain Dev. 2002;24:227–30. 10.1016/s0387-7604(02)00017-7. 12015165 10.1016/s0387-7604(02)00017-7

[53] Langkau N, Martin N, Brandt R, Zügge K, Quast S, Wiegele G, et al. TSC1 and TSC2 mutations in tuberous sclerosis, the associated phenotypes and a model to explain observed TSC1/TSC2 frequency ratios. Eur J Pediatr. 2002;161:393–402. 10.1007/s00431-001-0903-7. 12111193 10.1007/s00431-001-0903-7

[54] Li W, Zhou P, Zhao C, Zhang Y. A novel de novo mutation in the TSC2 gene in a Chinese patient with tuberous sclerosis complex. J Neurogenet. 2016;30:285–7. 10.1080/01677063.2016.1242585. 27776463 10.1080/01677063.2016.1242585

[55] He J, Zhou W, Shi J, Lin J, Zhang B, Sun Z. TSC1 and TSC2 gene mutations in chinese tuberous sclerosis complex patients clinically characterized by epilepsy. Genet Test Mol Biomark. 2020;24:1–5. 10.1089/gtmb.2019.0094. 10.1089/gtmb.2019.0094 31855466

[56] Glushkova M, Bojinova V, Koleva M, Dimova P, Bojidarova M, Litvinenko I, et al. Molecular genetic diagnostics of tuberous sclerosis complex in Bulgaria: six novel mutations in the TSC1 and TSC2 genes. J Genet. 2018;97:419–27. 29932062

[57] Hagon-Nicod O, Fellmann F, Novy J, Lebon S, Wider C, Lazor R, et al. Tuberous sclerosis: a survey in the canton of Vaud, Switzerland. Front Med. 2024;11:1513619. 10.3389/fmed.2024.1513619. 10.3389/fmed.2024.1513619 PMC11669681 39726678

[58] Praticò AD, Di Napoli C, Salafia S, Dammino E, Piccione M, Calì F, et al. Genetic screening of tuberous sclerosis complex in Sicily with a focus on neurological manifestations. Sci Rep. 2025;15:20347. 10.1038/s41598-025-04718-6. 40579409 10.1038/s41598-025-04718-6 PMC12205088

[59] Almuqbil M, Aldoohan W, Alhinti S, Almahmoud N, Abdulmajeed I, Alkhodair R, et al. Review of the spectrum of tuberous sclerosis complex: The Saudi Arabian Experience. Neurosciences. 2024;29:113–21. 10.17712/nsj.2024.2.20230061. 38740395 10.17712/nsj.2024.2.20230061 PMC11305360

[60] Dulamea AO, Arbune AA, Anghel D, Boscaiu V, Andronesi A, Ismail G. Neurological and dermatological manifestations of tuberous sclerosis complex: report from a romanian tertiary hospital cohort. J Clin Med. 2023;12:6550. 10.3390/jcm12206550. 37892688 10.3390/jcm12206550 PMC10607726

[61] Pearsson K, Björk Werner J, Lundgren J, Gränse L, Karlsson E, Källén K, et al. Childhood tuberous sclerosis complex in southern Sweden: a paradigm shift in diagnosis and treatment. BMC Pediatr. 2023;23:329. 10.1186/s12887-023-04137-4. 37386496 10.1186/s12887-023-04137-4 PMC10308728

[62] Gupta A, De Bruyn G, Tousseyn S, Krishnan B, Lagae L, Agarwal N, et al. Epilepsy and neurodevelopmental comorbidities in tuberous sclerosis complex: a natural history study. Pediatr Neurol. 2020;106:10–6. 10.1016/j.pediatrneurol.2019.12.016. 32139167 10.1016/j.pediatrneurol.2019.12.016

[63] Tye C, Mcewen FS, Liang H, Underwood L, Woodhouse E, Barker ED, et al. Long-term cognitive outcomes in tuberous sclerosis complex. Dev Med Child Neurol. 2020;62:322–9. 10.1111/dmcn.14356. 31538337 10.1111/dmcn.14356 PMC7027810

[64] Eser M, Hekimoglu G, Kutlubay B, Sager SG, Turkyilmaz A. Analysis of TSC1 and TSC2 genes and evaluation of phenotypic correlations with tuberous sclerosis. Mol Genet Genomics. 2025;300:6. 10.1007/s00438-024-02210-w. 10.1007/s00438-024-02210-w 39722056

[65] Kim H, Lee SR, Shin HJ, Jang S, Kim SH, Lee JS, et al. Clinical manifestations and treatments of patients with tuberous sclerosis with subependymal giant cell astrocytoma. Pediatr Neurol. 2025;166:1–6. 10.1016/j.pediatrneurol.2025.02.003. 40020251 10.1016/j.pediatrneurol.2025.02.003

[66] Yang G, Shi ZN, Meng Y, Shi XY, Pang LY, Ma SF, et al. Phenotypic and genotypic characterization of Chinese children diagnosed with tuberous sclerosis complex. Clin Genet. 2017;91:764–8. 10.1111/cge.12920. 27859028 10.1111/cge.12920

[67] Davis PE, Filip-Dhima R, Sideridis G, Peters JM, Au KS, Northrup H, et al. Presentation and diagnosis of tuberous sclerosis complex in infants. Pediatrics. 2017;140:e20164040. 10.1542/peds.2016-4040. 29101226 10.1542/peds.2016-4040 PMC5703775

[68] Ogórek B, Hamieh L, Hulshof HM, Lasseter K, Klonowska K, Kuijf H, et al. TSC2 pathogenic variants are predictive of severe clinical manifestations in TSC infants: results of the EPISTOP study. Genet Med. 2020;22:1489–97. 10.1038/s41436-020-0823-4. 32461669 10.1038/s41436-020-0823-4

[69] Bittles AH, Black ML. Consanguinity, human evolution, and complex diseases. Proc Natl Acad Sci. 2010;107:1779–86. 10.1073/pnas.0906079106. 19805052 10.1073/pnas.0906079106 PMC2868287

[70] Seliem MA, Bou-Holaigah IH, Al-Sannaa N. Influence of consanguinity on the pattern of familial aggregation of congenital cardiovascular anomalies in an outpatient population. Public Health Genomics. 2007;10:27–31. 10.1159/000096277. 10.1159/000096277 17167247

[71] Kozlowski P, Roberts P, Dabora S, Franz D, Bissler J, Northrup H, et al. Identification of 54 large deletions/duplications in TSC1 and TSC2 using MLPA, and genotype-phenotype correlations. Hum Genet. 2007;121:389–400. 10.1007/s00439-006-0308-9. 17287951 10.1007/s00439-006-0308-9

[72] Lyczkowski DA, Conant KD, Pulsifer MB, Jarrett DY, Grant PE, Kwiatkowski DJ, et al. Intrafamilial phenotypic variability in tuberous sclerosis complex. J Child Neurol. 2007;22:1348–55. 10.1177/0883073807307093. 18174550 10.1177/0883073807307093

[73] Purbasari U, Prihartono NA, Helda N, Audita FR, Dharmawan BS. Challenges of siblings with tuberous sclerosis showing various manifestations and severe complications. Radiol Case Rep. 2024;19:2566–73. 10.1016/j.radcr.2024.03.002. 38596180 10.1016/j.radcr.2024.03.002 PMC11001616

[74] Kotulska K, Kwiatkowski DJ, Curatolo P, Weschke B, Riney K, Jansen F, et al. Prevention of epilepsy in infants with tuberous sclerosis complex in the EPISTOP trial. Ann Neurol. 2021;89:304–14. 10.1002/ana.25956. 33180985 10.1002/ana.25956 PMC7898885

[75] Gahl WA, Wong-Rieger D, Hivert V, Yang R, Zanello G, Groft S. Essential list of medicinal products for rare diseases: recommendations from the IRDiRC rare disease treatment access working group. Orphanet J Rare Dis. 2021;16:308. 10.1186/s13023-021-01923-0. 34256816 10.1186/s13023-021-01923-0 PMC8278724

[76] Marques R, Belousova E, Benedik MP, Carter T, Cottin V, Curatolo P, et al. Treatment patterns and use of resources in patients with tuberous sclerosis complex: insights from the TOSCA registry. Front Neurol. 2019;10:1144. 10.3389/fneur.2019.01144. 31708865 10.3389/fneur.2019.01144 PMC6823684

