# Supplementary material for: Genotypic and phenotypic features of 23 Egyptian patients with tuberous sclerosis complex
Source: BMC Pediatr. 2026 Jun 10;26:561. doi: 10.1186/s12887-026-07095-9 (PMC13255346; doi:10.1186/s12887-026-07095-9)
Supplement: Supplementary file 2 — Additional file 2: Characteristics of seizures in 20 patients with tuberous sclerosis complex. [file 12887_2026_7095_MOESM2_ESM.docx]

**Additional file 2** Characteristics of seizures in 20 patients with tuberous sclerosis complex

| Patient ID**^*^** | Age at onset | Seizure type | EEG characteristics | Antiseizure medications | Treatment response |
| --- | --- | --- | --- | --- | --- |
|  |  |  |  |  |  |
| 1 | 3 mo | Epileptic spasm, focal | Hypsarrhythmia, multifocal epileptiform discharge | Vigabatrin, levetiracetam | Partial |
| 2 | 2 mo | Epileptic spasm, focal | Hypsarrhythmia, multifocal epileptiform discharge | Vigabatrin, ACTH, sodium valproate, topiramate, clonazepam | Drug-resistant |
| 3 | 18 mo | Focal, generalized myoclonic | Focal epileptiform discharge, generalized polyspikes | Levetiracetam, oxcarbazepine, topiramate, clonazepam | Drug-resistant |
| 4 | 2 y | Focal, generalized myoclonic | Multifocal epileptiform discharge | Levetiracetam, oxcarbazepine | Partial |
| 5 | 3 mo | Epileptic spasm, focal | Hypsarrhythmia, focal epileptiform discharge | Vigabatrin, ACTH, levetiracetam | Partial |
| 6 | 1 mo | Epileptic spasm, focal | Hypsarrhythmia, multifocal epileptiform discharge | Vigabatrin, ACTH, levetiracetam, topiramate | Drug-resistant |
| 7 | 4 mo | Epileptic spasm, focal | Hypsarrhythmia, multifocal epileptiform discharge | Vigabatrin, levetiracetam | Partial |
| 8 | 8 mo | Epileptic spasm, focal, generalized tonic-clonic | Multifocal epileptiform discharge, generalized spike-wave discharge | Vigabatrin, ACTH, sodium valproate, levetiracetam | Drug-resistant |
| 11 | 2.2 y | Generalized myoclonic, tonic-clonic | Multifocal epileptiform discharge, generalized polyspikes | Levetiracetam, sodium valproate | Partial |
| 12 | 6 mo | Epileptic spasm, focal | Hypsarrhythmia, multifocal epileptiform discharge | Vigabatrin, levetiracetam, oxcarbazepine | Partial |
| 14 | 3 mo | Epileptic spasm, focal | Hypsarrhythmia, focal epileptiform discharge | Vigabatrin, levetiracetam, oxcarbazepine | Partial |
| 15 | 15 d | Epileptic spasm, focal, generalized myoclonic | Hypsarrhythmia, multifocal epileptiform discharge, generalized polyspikes | Vigabatrin, ACTH, levetiracetam, sodium valproate, topiramate, clonazepam | Drug-resistant |
| 16 | 2 y | Focal, generalized | Multifocal epileptiform discharge | Oxcarbazepine, levetiracetam | Partial |
| 17 | 3 y | Generalized myoclonic | Generalized polyspikes | Sodium valproate, levetiracetam | Partial |
| 18 | 8 mo | Epileptic spasm, focal | Hypsarrhythmia, focal epileptiform discharge | Vigabatrin, ACTH, levetiracetam, oxcarbazepine | Drug-resistant |
| 19 | 2 y | Focal | Multifocal epileptiform discharge | Oxcarbazepine, levetiracetam | Partial |
| 20 | 6 mo | Epileptic spasm, focal | Hypsarrhythmia, focal epileptiform discharge | Vigabatrin, ACTH, levetiracetam, oxcarbazepine, clonazepam | Drug-resistant |
| 21 | 4 mo | Epileptic spasm, focal | Hypsarrhythmia, multifocal epileptiform discharge | Vigabatrin, ACTH, levetiracetam, topiramate | Drug-resistant |
| 22 | 2 y | Focal, generalized myoclonic | Multifocal epileptiform discharge, generalized polyspikes | Levetiracetam, sodium valproate | Partial |
| 23 | 2 y | Focal | Multifocal epileptiform discharge | Oxcarbazepine, levetiracetam, | Partial |

**^*^** Patients 9, 10, and 13 displayed no clinical seizures

– , absent; +, present; ACTH, adrenocorticotropic hormone; mo, month; y, year
