# Supplementary material for: Genotypic and phenotypic features of 23 Egyptian patients with tuberous sclerosis complex
Source: BMC Pediatr. 2026 Jun 10;26:561. doi: 10.1186/s12887-026-07095-9 (PMC13255346; doi:10.1186/s12887-026-07095-9)
Supplement: Supplementary file 3 — Additional file 3: Comparison between patients with TSC2 and TSC1 variants. [file 12887_2026_7095_MOESM3_ESM.docx]

**Additional file 3** Comparison between patients with *TSC2* and *TSC1* variants

| Feature | Number (%) / median | |
| --- | --- | --- |
|  | *TSC2*  *(n=21)* | *TSC1*  *(n=2)* |
| Males/females | 14/7 | 2/0 |
| Current age (year) | 8.7 | 5.5 |
| Age at onset (month) | 8 | 24 |
| Presenting feature |  |  |
| Seizures | 17 (81%) | 2 (100%) |
| Developmental delay | 4 (19%) | 0 |
| Neurological features |  |  |
| Seizures | 18 (86%) | 2 (100%) |
| TANDs | 18 (86%) | 0 |
| Brain imaging |  |  |
| Cortical tubers | 20 (95%) | 2 (100%) |
| SENs | 17 (81%) | 2 (100%) |
| SEGA | 1 (5%) | 0 |
| Skin manifestations |  |  |
| Hypomelanotic macules | 21 (100%) | 2 (100%) |
| Facial angiofibroma | 14 (67%) | 1 (50%) |
| Shagreen patch | 11 (52%) | 0 |
| Ungual fibroma | 8 (38%) | 0 |
| Oral fibroma | 6 (29%) | 0 |
| Extra-neurocutaneous features |  |  |
| Renal AMLs | 10 (48%) | 0 |
| Renal cysts | 6 (29%) | 0 |
| Cardiac rhabdomyoma | 8 (38%) | 1 (50%) |
| Hepatic tumors | 5 (24%) | 0 |
| Retinal hamartoma**^*^** | 4 (25%) | 0 |
| Bone anomalies**^*^** | 2 (12.5%) | 0 |
| Dental anomalies | 2 (10%) | 0 |
| Pulmonary LAM | 0 | 0 |

**^*^** Assessed in only 18 out of the 23 patients

AML, Angiomyolipoma; LAM: Lymphangioleiomyomatosis; SEGA, subependymal giant cell astrocytoma; SENs, subependymal nodules; TANDs, Tuberous sclerosis complex-associated neuropsychiatric disorders
