# Supplementary material for: Genotypic and phenotypic features of 23 Egyptian patients with tuberous sclerosis complex
Source: BMC Pediatr. 2026 Jun 10;26:561. doi: 10.1186/s12887-026-07095-9 (PMC13255346; doi:10.1186/s12887-026-07095-9)
Supplement: Supplementary file 4 — Additional file 4: Novel in silico prediction of splicing mechanism for TSC2 c.2098G>A variant identified in Case 16. [file 12887_2026_7095_MOESM4_ESM.docx]

**Additional file 4** Novel in silico prediction of splicing mechanism for TSC2 c.2098G>A variant identified in Case 16

**
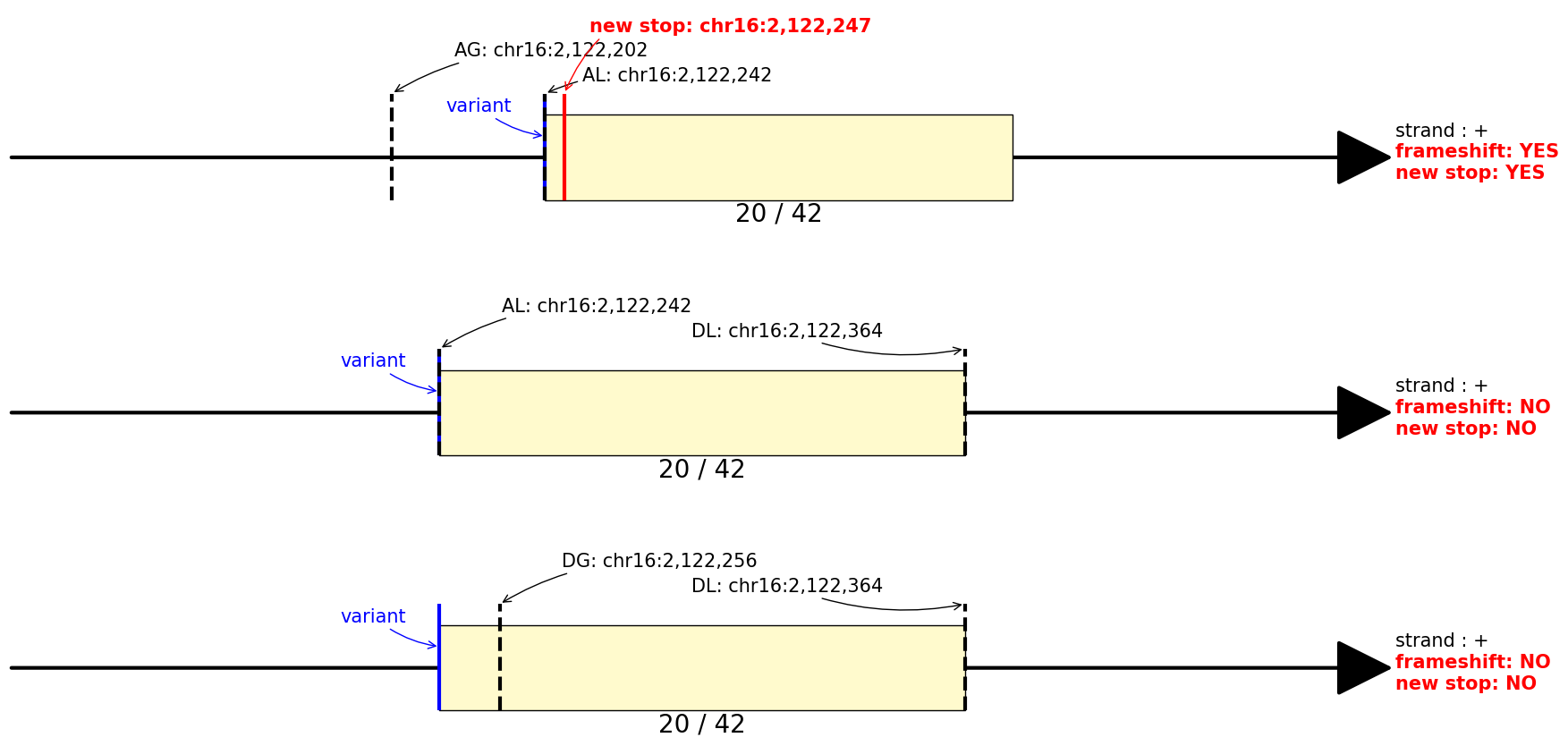
**

This schematic presentation depicts potential splicing changes due to TSC2 c.2098G>A variant. Although Yamamoto et al. (2002) reported this variant and suggested potential exon skipping, our *in silico* prediction using spliceAI indicates that the *TSC2* c.2098G>A variant is most likely to cause intron retention of 40 nucleotides adjacent to exon 20, rather than exon skipping. While this variant is expected to result in loss of function, current guidelines of the American College of Medical Genetics and Genomics (ACMG) and the Association for Molecular Pathology (AMP) would require experimental evidence of splicing impact, such as cDNA sequencing, RNA sequencing, or a minigene assay, to support reclassification from a variant of uncertain significance to likely pathogenic.

Yamamoto T, Pipo JR, Feng J-H, Takeda H, Nanba E, Ninomiya H, et al. Novel TSC1 and TSC2 mutations in Japanese patients with tuberous sclerosis complex. Brain Dev. 2002;24: 227–230. doi:10.1016/s0387-7604(02)00017-7
